# The Effect of Transformational Leadership, Work Motivation and Culture on Millennial Generation Employees Performance of the Manufacturing Industry in the Digital Era

**DOI:** 10.3389/fpsyg.2022.908966

**Published:** 2022-05-09

**Authors:** Mahdani Ibrahim, Banta Karollah, Vilzati Juned, Mukhlis Yunus

**Affiliations:** ^1^Department of Management, Faculty of Economics, Universitas Syiah Kuala, Banda Aceh, Indonesia; ^2^Department of Management, Sekolah Tinggi Ilmu Ekonomi Sabang, Banda Aceh, Indonesia

**Keywords:** organizational culture, transformational leadership, performance, motivation, millennial generation

## Introduction

In this era of the industrial revolution, the future challenges in the industry must be to improve infrastructure development and adequate employee performance. On the other hand, the company is also getting a demographic bonus in the form of the number of employees of the millennial generation who should be of a productive age and able to support the company's performance. The success of an organization is determined by its ability to manage various kinds of resources, one of which is very important, namely human resources. According to Suprapti et al. (2020) and Sunarsi et al. (2021), employees are always attached to any organizational resource as a determining factor for their existence and role in contributing to the achievement of organizational goals effectively and efficiently faced by companies in the coming year will be even more difficult. The Central Statistics Agency (BPS) shows that 50% of workers in Indonesia are <30 years old, which means that millennials are active workers who dominate human resources in an organization. According to Sun and Leithwood (2015) the millennial generation is hope for the company. To have human resources that support the progress of the organization requires high motivation from employees in improving performance. Employee motivation is influenced by the work atmosphere that supports its performance.

In several studies, organizational culture is present in providing an indirect influence on employee performance. According to Chan et al. (2019) culture is an important part of the internal environment of an organization. Organizational culture is a set of values, beliefs, behaviors, customs, and attitudes that help members of the organization understand and translate the attitudes that must be taken in dealing with something. Organizational culture plays an important role in influencing the level of motivation of employees to carry out their work. According to Charoensukmongkol and Puyod (2021) and Dewi et al. (2022), there is a clear dependence between organizational culture and employee motivation, where organizational culture must be encouraged to increase employee performance motivation. In addition to a supportive work environment, organizations must have leaders who are able to motivate their members to achieve a goal. Leadership has a big influence on an achievement in the organization. The quality of a leader influences its members to build self-confidence, motivation, and commitment to achieve company goals. According to Direction (2015) and Dewi et al. (2022) motivation becomes very important as a factor in achieving employee performance. Motivation affects a person or individual to be involved in activities and work that leads to goals as satisfaction. With this motivation employees have From this background, researchers feel that to improve the performance of millennial employees, research is needed on the relationship between organizational culture and transformational leadership in influencing the performance of next-generation employees millennials through motivation.

### Research Gap of This Research

#### Relationship Between Transformational Leadership Variables and Performance

Research by Chan et al. (2019) and Charoensukmongkol and Puyod (2021) stated that transformational leadership has a significant effect on performance. This result is not in line with research by Direction (2015) and Dewi et al. (2022) state that transformational leadership has no significant effect on performance.

#### The Relationship Between Work Motivation and Performance Variables

Research by Kadiyono et al. (2020) and Muliati et al. (2022) stated that work motivation has a significant effect on performance. This result is not in line with research by Noruzy et al. (2013) and Nguyen and Luu (2019) stated that work motivation had no significant effect on performance.

#### Relationship Between Work Culture and Performance Variables

Research by Prayuda (2019) and Purwanto et al. (2021) stated that work motivation has a significant effect on performance. This result is not in line with research by Singgih et al. (2020) and Sunarsi et al. (2021) state that work motivation has no significant effect on performance.

## Methods

This study uses quantitative methods through surveys, data from online questionnaires are analyzed using an alternative analysis technique Partial Least Square (PLS) using SmartPLS 3.0 software. Primary data was obtained from filling out online questionnaires to 500 respondents from Millennial Generation Employees in the Manufacturing Industry in the Digital Era determined by simple random sampling method.

**Figure 1:**
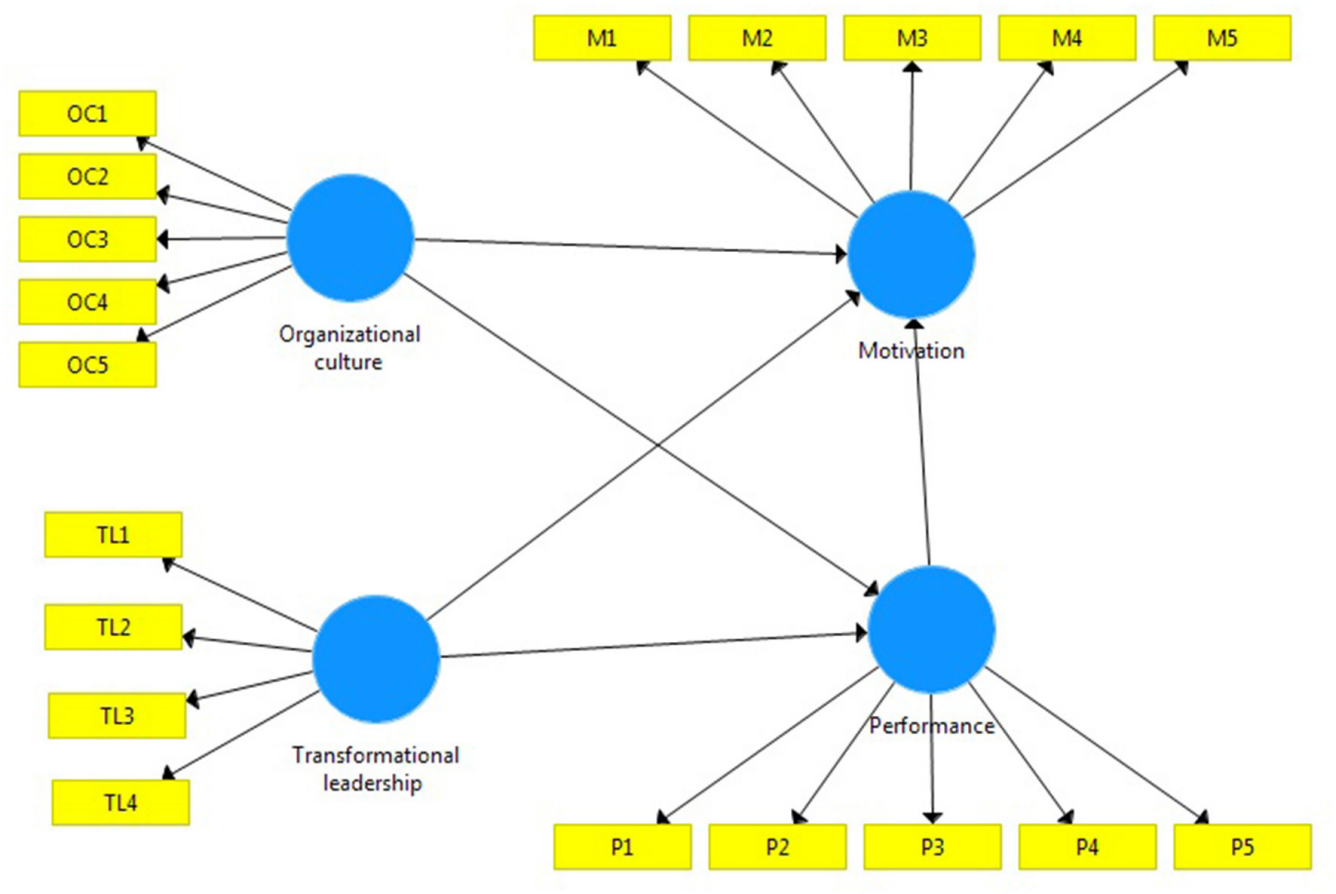
Research model.

The hypothesis that the researcher proposes is as follows:

H1: Organizational culture has a significant effect on motivation. The hypothesis that the researcher proposes is as follows in Figure 1.

H2: Transformational leadership has a positive and positive effect significant to motivation.

H3: Motivation has a significant positive effect on performance.

H4: Organizational culture has a significant positive effect on performance through motivation.

H5: Motivation mediates the relationship between transformational leadership and performance.

## Result And Discussion

### Based on the Results of Data Processing, the Results Obtained

#### Hypothesis 1: Organizational Culture Has a Significant Effect on Motivation

The path coefficient of the influence of Organizational Culture on motivation is 0.432 with a t-statistic of 5.876 which is much larger than the t-table value of 1.96. This can be interpreted that there is a significant positive effect on the relationship between Organizational Culture and Motivation. So, the first hypothesis in this study is accepted.

#### Hypothesis 2: Transformational Leadership Has a Positive and Significant Effect on Motivation

The path coefficient of the effect of Transformational Leadership on Motivation is 0.432 with a t-statistic of 4.762 which is greater than the t-table value of 1.96. This can be interpreted that there is a significant positive effect on the relationship of transformational leadership to motivation. It can be interpreted that transformational leadership felt by employees is able to provide motivation within employees.

#### Hypothesis 3: Motivation Has a Significant Positive Effect on Performance

The path coefficient of the effect of motivation on performance is 0.873 with a t-statistic of 6.872 which is greater than the t-table value of 1.96. This means that there is a significant positive effect on the relationship between motivation and performance. It can be interpreted that employees really need motivation to achieve their performance.

#### Hypothesis 4: Organizational Culture Has a Significant Positive Effect on Performance Through Motivation

Based on the data analysis, it is known that in the analysis of the mediating relationship the VAF value raised is 0.983. This value indicates a significant result of the mediating relationship between organizational culture and performance through motivation. Organizational culture has an influence on employee motivation, and has a direct influence on employee performance. So, hypothesis 4 in this study is accepted.

#### Hypothesis 5: Motivation Mediates the Relationship Between Transformational Leadership and Performance

The path coefficient of the effect of transformational leadership on performance is 0.873 with a t-statistic of 0.605 which is smaller than the t-table value of 1.96. This figure means that there is a significant relationship. To find out the results of the mediation relationship, a significant direct relationship value is needed. So, hypothesis 5 in this study is accepted.

## Discussion

### Organizational Culture on Motivation

Based on the research results, organizational culture is proven to have a significant positive effect on motivation. This means that the organizational culture formed by the company will have a positive and significant influence on employee motivation. This is in line with the opinion of Robbins (2013), namely that organizational culture functions as a meaning-making and control mechanism that shapes employee attitudes and behavior. According to Chan et al. (2019) and Charoensukmongkol and Puyod (2021) argue that organizational culture refers to the system of meaning possessed by team members that distinguishes the organization from other organizations. In accordance with the results of research conducted by Sun and Leithwood (2015) and Dewi et al. (2022) that when companies develop organizational culture, the higher the level of employee motivation.

### Transformational Leadership on Motivation

The results of this study regarding transformational leadership on motivation found that transformational leadership has a significant positive effect on motivation. Transformational leadership gives confidence to employees that they can achieve results, this is one form of motivation received by employees. According to the results of the questionnaire filled in by the respondents, according to Prayuda (2019), Singgih et al. (2020), Suprapti et al. (2020), Purwanto et al. (2021), and Sunarsi et al. (2021), employees feel effectively facilitated to carry out learning that occurs in the organization. This is because transformational leaders are able to mobilize existing resources to complement, strengthen, and improve the quality of everyone in the organization to be involved in achieving goals. Optimization The motivation given by transformational leaders can practically be in the form of giving assignments, jobs, work targets that are really challenging and provide opportunities for every organization to think creatively both in providing proposals, problem solving, and decision making. Each individual according to needs, abilities, and aspirations that can differ from transformational leaders m encourage employees to be motivated in completing their tasks. In accordance with the results of research conducted by Sun and Leithwood (2015); Chan et al. (2019); Charoensukmongkol and Puyod (2021), and Dewi et al. (2022) that the hypothesis regarding transformational leadership and its effect on motivation in employees take place effectively.

### Motivation Toward Performance

Based on the results of the study, motivation is proven to have a significant positive effect on performance. Employees are ordinary people who cannot be separated from insecurity, despair, giving up even though they have not struggled. According to Noruzy et al. (2013) and Nguyen and Luu (2019), these feelings can make employees fail to achieve their expectations or make their performance stop in the middle of the road. This condition can be overcome with motivation from within themselves, the surrounding environment, and inspiration from other people. The motivation that arises in employees makes the enthusiasm to work increase. With increased motivation, employee performance will also increase. According to Kadiyono et al. (2020) and Muliati et al. (2022) suggested that employees who motivated to help the company to achieve its goals. For employees who are motivated, time will feel fast when they are in the work environment because they enjoy the process of doing their job so that there is a willingness to do work. Employees who are motivated can be motivated by a desire to achieve a goal. Respondents have encouragement to achieve the maximum level of success in ta targets, goals, and job criteria given to them.

### Organizational Culture on Performance Through Motivation

Based on the results in this study, it was found that organizational culture on performance through motivation had no significant effect. In testing the hypothesis, the direct relationship between organizational culture and performance has a significant effect, as can be seen from the value of the hypothesis test obtained in chapter V. However, when testing the mediating relationship between organizational culture and performance through motivation, an insignificant relationship was found. The same results were also found in the results of research conducted by Nguyen and Luu (2019) where the motivational variable did not significantly mediate the relationship between organizational culture and performance. In the research of Noruzy et al. (2013), Nguyen and Luu (2019), Kadiyono et al. (2020), and Muliati et al. (2022) also said that the relationship between organizational culture and employee performance is considered to be indirectly mediated by employee motivation. When viewed from the distribution of answers made by respondents on question points regarding competition in producing work optimally has the highest neutral value from the other questions. This can mean that there are some respondents who are not sure of the motivational drive that comes from organizational culture to improve performance. According to Nguyen and Luu (2019) conducted a study who found an insignificant mediating relationship on organizational culture on performance through motivation. According to Kadiyono et al. (2020) and Muliati et al. (2022) each individual is typically different from one another. Inequality includes needs, desires, interests, values, attitudes, and accepted norms. So it is natural when they have different needs to motivate themselves to achieve good performance. It seems that in this case in addition to taking the company's core values, efforts are needed to unite the characteristics or personalities that vary among employees into a stronger organizational culture.

### Motivation Mediates the Relationship Between Transformational Leadership and Performance

Based on the results of this study, it was found that the mediating relationship between transformational leadership and performance through motivation was found to be an insignificant relationship. The results of the hypothesis analysis found that leadership has an insignificant relationship to performance. Meanwhile, to analyze the mediation relationship, a significant direct relationship is needed. So in this case the analysis of the mediation relationship cannot be carried out. The results of this study are in accordance with the results of research conducted by Suprapti et al. (2020) and Sunarsi et al. (2021) namely that work motivation does not significantly mediate the relationship between transformational leadership and performance, and also research conducted by Suprapti et al. (2020) and Sunarsi et al. (2021) who found an insignificant mediating relationship between transformational leadership to performance through motivation as a mediating variable. In the direct analysis, transformational leadership significantly positively affects employee motivation. That is, the existence of transformational leadership means that transformational leadership has a positive effect on employee motivation. Employee motivation is increasing but it turns out that this has no impact on their performance. Back to the needs of each individual for their background and work achievement goals, they have their own needs to achieve good performance including remuneration, career development and recognition.

### Practical and Theoretical Contribution of Research

Transformational leadership has a positive and significant effect on the motivation, these results are in line with research by Chan et al. (2019) and Charoensukmongkol and Puyod (2021) stated that transformational leadership has a significant effect on performance. This result is not in line with research by Direction (2015) and Dewi et al. (2022) state that transformational leadership has no significant effect on performance. Motivation has a significant positive effect on the performance, this result is in line with research by Kadiyono et al. (2020) and Muliati et al. (2022) stated that work motivation has a significant effect on performance. This result is not in line with research by Noruzy et al. (2013) and Nguyen and Luu (2019) stated that work motivation had no significant effect on performance. Organizational culture had a significant effect on Millennial generation employee motivation, this result is in line with research by Prayuda (2019) and Purwanto et al. (2021) stated that work motivation has a significant effect on performance. This result is not in line with research by Singgih et al. (2020) and Sunarsi et al. (2021) state that work motivation has no significant effect on performance.

## Conclusion

Based on the results of data analysis in this study, it can be concluded that organizational culture has a significant effect on millennial generation employee motivation, transformational leadership has a positive and significant impact on millennial generation employee motivation, motivation has a significant positive effect on millennial generation employee performance, organizational culture has a significant positive effect on employee motivation. not significant to the performance of millennial employees, motivation mediates the relationship between transformational leadership and the performance of millennial employees not significantly. Suggestions given by researchers include practical advice and academic advice. As a practical suggestion, researchers give advice to companies to create an organizational culture that is oriented toward employee motivation so that companies can map things that can be behind the emergence of motivation in employees, especially in the generation that dominates today, namely the millennial generation. Although the result of the path coefficient value is positive and significant, but it is classified as a fairly low number, it is necessary to study the motivational content in organizational culture. Academic advice from researchers is that for further research, further discussion and analysis can be carried out on the factors that affect employee performance because in this study researchers are still limited to using only two variables as X variables which are adjusted to the needs of the respondents. The novelty of this research is a new model of the relationship between the variables of Transformational leadership, work motivation, culture and employee performance of the millennial generation in the manufacturing industry in the digital era.

## Author Contributions

MI conceived the presented idea. BK developed the theory and performed the computations. VJ verified the analytical methods. MI encouraged BK and VJ to investigate topic and supervised the findings of this work. All authors discussed the results and contributed to the final manuscript.

## Conflict of Interest

The authors declare that the research was conducted in the absence of any commercial or financial relationships that could be construed as a potential conflict of interest.

## Publisher's Note

All claims expressed in this article are solely those of the authors and do not necessarily represent those of their affiliated organizations, or those of the publisher, the editors and the reviewers. Any product that may be evaluated in this article, or claim that may be made by its manufacturer, is not guaranteed or endorsed by the publisher.

## References

[B1] Chan S. W. Ang S. F. Andleeb N. Ahmad M. F. Zaman I. (2019). The influence of transformational leadership on organization innovation in Malaysian manufacturing industry. Int. J. Supply Chain Manag. 8, 971–976. 10.31838/srp.2019.10.176

[B2] Charoensukmongkol P. Puyod J. V. (2021). Influence of transformational leadership on role ambiguity and work–life balance of Filipino University employees during COVID-19: does employee involvement matter? Int. J. Leadership Educ. 4, 1–20. 10.1080/13603124.2021.1882701

[B3] Dewi D. Y. Supriadi Y. N. Iswanto A. H. (2022). The effect of transformational leadership, quality of work-life on organizational citizenship behavior with organizational commitment mediation. J. Soc. Sci. 3, 308–323. 10.31838/srp.2022.10.123

[B4] Direction S. (2015). Transformational leadership: the impact of its behaviors on manufacturing strategy. Strat. Direct. 31, 25–27. 10.1108/SD-12-2014-0169

[B5] Kadiyono A. L. Sulistiobudi R. A. Haris I. Wahab M. K. A. Ramdani I. Purwanto A.. (2020). Develop leadership style model for indonesian teachers performance in Education 4.0 era. Syst. Rev. Pharmacy 11, 363–373. 10.31838/srp.2020.10.111

[B6] Muliati L. Asbari M. Nadeak M. Novitasari D. Purwanto A. (2022). Elementary school teachers performance: how the role of transformational leadership, competency, and self-efficacy?. Int. J. Soc. Manag. Stud. 3, 158–166. 10.31838/srp.2022.10.109

[B7] Nguyen T. T. N. Luu T. M. N. (2019). Linking transformational leadership and organizational performance: an empirical investigation of manufacturing firms in Vietnam. Econ. Sociol. 12, 170–191. 10.14254/2071-789X.2019/12-2/10

[B8] Noruzy A. Dalfard V. M. Azhdari B. Nazari-Shirkouhi S. Rezazadeh A. (2013). Relations between transformational leadership, organizational learning, knowledge management, organizational innovation, and organizational performance: an empirical investigation of manufacturing firms. Int. J. Adv. Manufact. Technol. 64, 1073–1085. 10.1007/s00170-012-4038-y

[B9] Prayuda R. (2019). The influence of transformational leadership, organizational climate, innovative behavior and employee engagement on industrial employee performance with job satisfaction in the digital era. J. Indust. Eng. Manag. Res. 1, 13–23. 10.7777/jiemar.v1i1a.251

[B10] Purwanto A. Purba J. T. Bernarto I. Sijabat R. (2021). Effect of transformational leadership, job satisfaction, and organizational commitments on organizational citizenship behavior. Inovbiz Jurnal Inovasi Bisnis 9, 61–69. 10.35314/inovbiz.v9i1.1801

[B11] Robbins S. (2013). Organisational Behaviour. Pearson Higher Education AU.

[B12] Singgih E. Iskandar J. Goestjahjanti F. S. Fahlevi M. Nadeak M. Fahmi K.. (2020). The role of job satisfaction in the relationship between transformational leadership, knowledge management, work environment and performance. Solid State Technol. 63, 56–65. 10.31838/srp.2020.10.161

[B13] Sun J. Leithwood K. (2015). Direction-setting school leadership practices: A meta-analytical review of evidence about their influence. Sch. Eff. Sch. Improv. 26, 499–523. 10.1080/09243453.2015.1005106

[B14] Sunarsi D. Paramarta V. Munawaroh A. R. Bagaskoro J. N. Evalina J. (2021). Effect of transformational, transactional leadership and job satisfaction: evidence from information technology industries. Inform. Technol. Industry 9, 987–996. 10.17762/itii.v9i1.232

[B15] Suprapti S. Asbari M. Cahyono Y. Mufid A. (2020). Leadership style, organizational culture and innovative behavior on public health center performance during Pandemic Covid-19. J. Indust. Eng. Manag. Res. 1, 76–88. 10.5555/ijosmas.v3i2.107

